# Non-Criteria Antiphospholipid Antibodies: Risk Factors for Endothelial Dysfunction in Women with Pre-Eclampsia

**DOI:** 10.3390/life10100241

**Published:** 2020-10-14

**Authors:** Lara Belmar Vega, Gema Fernández Fresnedo, Juan Irure Ventura, Victoria Orallo Toural, Milagros Heras Vicario, Juan Carlos Ruiz San Millán, Emilio Rodrigo, Marcos López Hoyos

**Affiliations:** 1 Nephrology Service, University Hospital Marqués de Valdecilla, 39008 Santander, Spain; gema.fernandezf@scsalud.es (G.F.F.); mmilagros.heras@scsalud.es (M.H.V.); juancarlos.ruiz@scsalud.es (J.C.R.S.M.); emilio.rodrigo@scsalud.es (E.R.); 2 Immunology Service, University Hospital Marqués de Valdecilla, 39008 Santander, Spain; juan.irure@scsalud.es (J.I.V.); marcos.lopez@scsalud.es (M.L.H.); 3 Obstetrics and Gynecology Service, University Hospital Marqués de Valdecilla, 39008 Santander, Spain; victoria.orallo@scsalud.es

**Keywords:** pre-eclampsia, antiphospholipid antibodies, antiphosphatidylserine/prothrombin antibodies, pulse wave velocity, ankle-brachial index

## Abstract

The association between unconventional antiphospholipid antibodies and pre-eclampsia in patients without thrombotic manifestations and its relationship with endothelial dysfunction after delivery has been studied poorly. We included 157 pregnant women, 122 of them having developed pre-eclampsia (56 non-severe and 66 severe). The determination of classical and unconventional, as well as pulse wave velocity and ankle-brachial index were performed at three months after delivery. The prevalence of unconventional antiphospholipid antibodies was 22.9% and 54.9% in patients included in control and pre-eclampsia groups, respectively (*p* = 0.001). The most frequent antiphospholipid antibody was IgM anti-phosphatidylserine/prothrombin in both cohorts. The presence of IgM anti-phosphatidylserine/prothrombin showed an association with the development of pre-eclampsia (OR = 5.4; CI 95% (2.0–14.9), *p* = 0.001) with an AUC of 0.744 (*p* < 0.001). Likewise, IgM anti-phosphatidylserine/prothrombin exhibited a positive linear correlation with pulse wave velocity values (rho = 0.830; *p* < 0.001) and an association with the presence of pulse wave velocity altered values (OR = 1.33; CI95% (1.10–1.59), *p* = 0.002). With regard to ankle braquial index values, the presence of IgM anti-phosphatidylserine/prothrombin displayed a weak negative correlation (rho = −0.466; *p* < 0.001) and an association with altered ankle braquial index values (OR = 1.08; CI 95% (1.04–1.13), *p* < 0.001). In patients who developed preeclampsia, the presence of IgM anti-phosphatidylserine/prothrombin could be associated with endothelial dysfunction, causing alteration of cardiovascular parameters.

## 1. Introduction

Pre-eclampsia (PE) is a hypertensive disorder of pregnancy, whose presentation includes the combination of maternal new-onset hypertension, which occurs most often after 20 weeks of gestation and frequently near term, and proteinuria, or evidence of other end-organ damage in the absence of proteinuria [1,2]. In pregnant women, this disease can cause a multisystem disease with central nervous system disturbances, liver, hematologic or renal dysfunction; and in the fetus, a fetal growth restriction with a potential risk of fetal death and forcing the pregnancy to end before term [3,4]. The pathophysiology of PE is not well understood. The main theories include genetic predisposition [5], loss of immunological tolerance between the fetoplacental unit and the maternal tissue, angiogenic imbalance, endothelial cell dysfunction, systemic inflammatory process, as well as alterations in the renin angiotensin aldosterone system and oxidative stress [6].

It is hypothesized that early-onset PE is mediated by the abnormal invasion of trophoblastic tissue into the maternal uterine wall, resulting in hypoperfusion and hypoxemia of the placenta and release of different cytokines and inflammatory factors in the maternal circulation that result in maternal endothelial damage [7,8]. On the other hand, in late-onset PE, the endothelial cell dysfunction has been linked to maternal “constitutional” factors, such as chronic hypertension, age, obesity or diabetes mellitus [9].

One of the main systemic autoimmune diseases associated with pregnancy morbidity and mortality is the antiphospholipid syndrome (APS) [10]. APS is a systemic autoimmune disorder defined by the persistent presence of antiphospholipid antibodies (aPL) in plasma of patients with vascular thrombosis and/or pregnancy morbidity. In its obstetric aspect, a patient is diagnosed with APS if she meets one or more of the clinical criteria, which include: one or more unexplained fetal deaths of morphologically normal fetus (normal fetal morphology confirmed by ultrasound or direct examination) at or beyond 10 weeks of gestation; one or more premature births of a morphologically normal neonate before the 34th week of gestation; prematurity must be secondary to eclampsia, severe preeclampsia, or placental insufficiency; or three or more consecutive spontaneous abortions before the 10th week of gestation after ruling out any anatomic or hormonal abnormalities in the mother and parental chromosomal causes. Together, these match the currently accepted Sydney criteria [11] with at least one of the following laboratory criteria, which require obtaining positive results in serum or plasma on two or more occasions separated by at least 12 weeks of lupus anticoagulant (LA), IgG and/or IgM anticardiolipin antibodies (aCL) measured by ELISA at medium or high titers or IgG and/or IgM anti-β2 glycoprotein antibodies I (aβ2GPI) measured by ELISA at medium or high titers. The presence of these autoantibodies has been associated with endothelial dysfunction [12,13,14].

However, there are some women with clinical signs that are highly suggestive of obstetric antiphospholipid syndrome (OAPS), but who are persistently negative for “criteria” aPL. This clinical entity is known as “seronegative OAPS” [15,16,17]. As a consequence of this seronegativity, several authors have suggested that testing for new aPL specificities [16,18] may help to more clearly identify OAPS in these patients. These “non-criteria” aPL include antibodies against prothrombin (aPT) and antibodies against the phosphatidylserine-prothrombin complex (aPS/PT) [19]. Although various studies indicate an association between aPS/PT and obstetric morbidity [20,21,22], suggesting that aPS/PT are an independent risk factor for obstetric complications stronger than classic aPL [23], other studies have not found this association [24,25,26]. Besides, the IgA aCL and antβi2GPI have been associated with thrombosis in renal transplantation [27], but their role in OAPS or obstetric manifestations is not clear. Due to these contradictory results, the presence of these autoantibodies have not been yet included in the diagnostic criteria of APS.

In recent years, several non-invasive techniques have been developed in order to evaluate the endothelial dysfunction of the peripheral blood vessels in an indirect manner, such as pulse wave velocity (PWV) and ankle-brachial index (ABI) [28]. PWV determination is a short-term non-invasive procedure, and simple to carry out in healthcare practice [29,30]. PWV is considered one of the most important organic damage markers with higher cardiovascular predictive value, higher reproducibility, and with an acceptable cost-effectiveness ratio [31,32,33]. There is evidence that in healthy pregnant women, the hemodynamic adaptations that occur during pregnancy determine a significant decrease of the PWV from the first to the second trimester of pregnancy, which increases at the beginning of the third trimester and returns to basal levels after delivery [34]. By contrast, in pregnant women who develop PE, a stronger vasoconstriction promotes an increase of the PWV throughout pregnancy and that even extends beyond delivery [35].

Moreover, the ankle-brachial index (ABI) is a non-invasive, inexpensive, simple technique that allows the evaluation of the risk of developing atherosclerosis. It has been demonstrated as a good predictor of peripheral vascular disease, as well as of stroke and cardiovascular events in middle-age and older populations, being at the same time an effective tool for the screening of subclinical atherosclerotic disease [36,37,38].

In the present study we aimed to highlight the relevance of unconventional aPLs and to establish their relationship with the appearance of abnormal parameters suggestive of endothelial dysfunction.

## 2. Results

### 2.1. Anthropometric Factors, Personal, Family and Obstetric History. Pregnancy Data

The main variables of control and PE groups are shown in Supplementary Table S1. No significant differences were observed in the mean age of pregnant women included in both cohorts. However, those who developed PE had higher prevalence of familial antecedents of hypertension and a lower percentage of the use of assisted reproductive techniques in comparison with the control group. Delivery in women belonging to the PE group occurred significantly earlier than in control group. For this reason, newborns of PE women had lower scores on the APGAR test, lower weight, and lower pH in blood drawn from the umbilical cord at birth time.

Eclampsia was diagnosed in only two women and HELLP syndrome in 16 women. Proteinuria was observed in 90.2% of the PE women and thrombopenia, alterations in liver and kidney function, were observed in 36.1%, 19.7% and 14.8%, respectively.

### 2.2. Association between Antiphospholipid Antibodies and Pre-Eclampsia

The prevalence of classical and unconventional aPL is shown in Table 1. Although there was no significant difference in the prevalence of classical aPLs between the control and the PE group, the rate of positivity for unconventional aPLs was significantly higher for the PE group, being IgM aPS/PT the aPL most related to PE. Of note, unconventional aPL and specifically IgM aPS/PT are more prevalent in the severe PE group than in the non-severe PE group.

The prevalence of classical versus unconventional aPL was identical in the control group, being 22.9% for both types of aPLs. Among classical aPL, IgG aCL was the most prevalent, whereas IgM aPS/PT was the most prevalent of the unconventional aPLs.

In the PE cohort, the prevalence of unconventional aPL (54.9%) was almost three times higher than the prevalence of classical aPL (18.9%). In this group of patients, the most prevalent classical aPL was IgG aβ2GPI (13.9%), and among unconventional aPL, IgM aPS/PT (47.5%). The post-hoc analysis showed that the study provided 80% of statistical power to detect differences ≥ 17% in the prevalence rates of antibodies between the pre-eclampsia and control groups. Regarding the severity of PE, patients that developed severe PE (S-PE) showed higher prevalence for all the aPLs that were analyzed (classical and unconventional), except for LA. Considering all the aPLs, IgM aPS/PT remained the most prevalent antibody in S-PE group (Table 1).

In the univariate logistic regression analysis, only IgM aPS/PT showed a significant association with the development of PE (OR = 5.4; 95% CI (2.0–14.9), *p* = 0.001). Likewise, when PE patients were divided according to the severity of the disease, this autoantibody was uniquely associated with the development of S-PE (OR = 4.4; 95% CI (2.0–9.4), *p* < 0.001) (Table 2).

**Figure 1:**
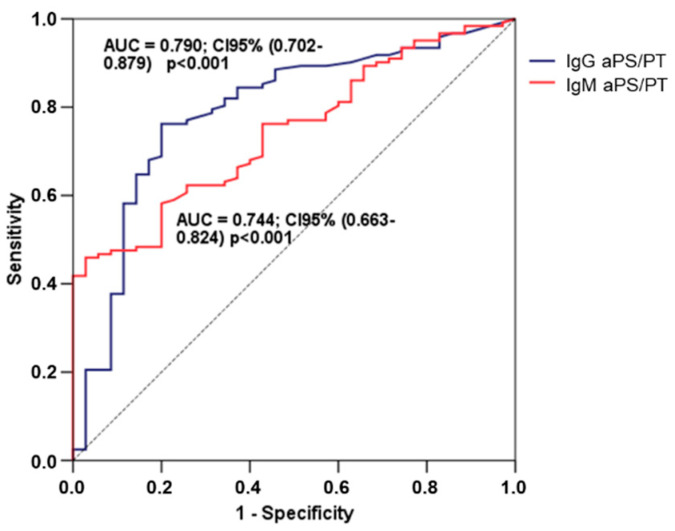
Receiver operating characteristic (ROC) curve analysis for the development of preeclampsia. Only the IgG and IgM aPS/PT antibodies presented statistically significant AUC (0.790 and 0.744, respectively).

According to receiver operating characteristic (ROC) curve analysis, none of the classical aPLS showed a statistically significant association with PE. Regarding unconventional antibodies, IgM aPS/PT reflected an AUC of 0.744 95% CI (0.663–0.824) (*p* < 0.001), with an optimal cut-off point established at 22.04 U/mL, (sensitivity = 62.3%; specificity = 74,3%; positive predictive value = 89.4% and negative predictive value = 36.1%) and the IgG aPS/PT showed an AUC of 0.790 95% CI (0.702–0.879) (*p* < 0.001), with an optimal cut-off point established at 2.78 U/mL, sensitivity = 76.2%; specificity = 80.0%; positive predictive value = 93.0% and negative predictive value = 49.1%) (Figure 1).

### 2.3. Main Parameters of Cardiovascular Involvement (PWV and ABI)

PWV was significantly higher in the PE group in comparison with control group (8.2 m/s; IQR (7.6–9.5) vs. 7.7 m/s; IQR (7.2–8.0), *p* < 0.001). Moreover, considering the severity of the PE, those patients who suffered S-PE showed higher levels of PWV (9.0 m/s; IQR (7.8–10.2)) than NS-PE group (8.0 m/s; IQR (7.6–8.9)), *p* = 0.015 (Table 3).

No linear correlation was observed between age and PWV (rho = 0.051; *p* = 0.527), nor between BMI and PWV (rho = 0.115; *p* = 0.150). Regarding other cardiovascular risk factors, no significantly differences were found in the levels of PWV between patients with or without dyslipidemia, diabetes mellitus or smoking habit.

Regarding ABI, the ABI values were significantly lower in patients who developed PE (1.04; IQR (0.90–1.26)) than in the control group (1.16; IQR (1.00–1.30)), *p* < 0.001. Likewise, considering the severity of the PE, those patients who suffered S-PE showed lower values of ABI (0.94; IQR (0.89–1.24)) than NS-PE group (1.11; IQR (0.97–1.36)), *p* < 0.001 (Table 3). No linear correlation was observed between age and ABI (rho = 0.004; *p* = 0.964), nor between BMI and ABI (rho = 0.177; *p* = 0.027). With respect to other cardiovascular risk factors, no significant differences were found in the values of ABI between patients with or without dyslipidemia or smoking habit. However, patients who presented diabetes mellitus had significantly lower ABI values than those patients without the disease (1.10; IQR (0.91–1.20) vs. 1.25; IQR (1.23–1.28)), *p* = 0.027

### 2.4. Relationship between aPLs and Cardiovascular Parameters

**Figure 2:**
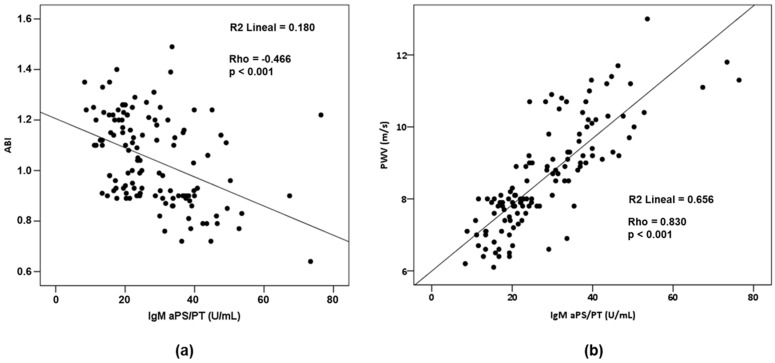
(**a**) Linear correlation between IgM aPS/PT and ankle brachial index (ABI). (**b**) Linear correlation between IgM aPS/PT and pulse wave velocity (PWV).

In the control group, the presence of IgM aCL and IgM aPS/PT showed a positive linear correlation with PWV (rho = 0.510; *p* = 0.002 and rho = 0.466; *p* = 0.005, respectively). However, none of the aPLs showed a linear significant correlation with the ABI. On the other hand, considering those women who developed PE, the presence of IgM aPS/PT showed an intense positive linear correlation with PWV (rho = 0.830; *p* < 0.001) and a weak negative correlation with ABI (rho = −0.466; *p* < 0.001) (Figure 2a,b).

In the control group, the univariate logistic regression analysis showed no significant association between classical or unconventional aPL and the presence of PWV values higher than the reference value established for the corresponding age group. In the same way, no significant association was observed with ABI values suggestive of vascular pathology.

Concerning the PE group, the univariate logistic regression analysis showed that IgM aPS/PT (OR = 1.33; CI 95% (1.10–1.59), *p* = 0.002) was significantly associated with the presence of PWV values higher than the reference value established for the corresponding age group (Supplementary Table S2). In the same way, no significant association was observed with ABI values suggestive of vascular pathology. For its part, only IgA aβ2GPI (OR = 1.08; CI 95% (1.01–1.15), *p* = 0.023) and IgM aPS/PT (OR = 1.08; CI 95% (1.04–1.13), *p* ≤0.001) showed significant association with ABI values suggestive of vascular pathology.

In the multivariate logistic regression analysis, the initial models included the different variables that were identified as potential predictors in the univariate regression analysis, as well as the classical cardiovascular risk factors (age, BMI, hypertension, dyslipidemia, diabetes, smoking). In the resulting models, IgM aPS/PT (OR = 1.37; CI 95%: 1.10–1.72; *p* = 0.006) was associated with the presence of altered PWV values. Moreover, IgA aβ2GPI (OR = 1.10 CI 95% (1.02–1.18)) and IgM aPS/PT (OR = 1.09; CI 95% (1.04–1.14) were associated with the presence of altered ABI values, *p* = 0.012 and *p* < 0.001, respectively (Table 4).

## 3. Discussion

Our main finding was that unconventional aPL was specifically related to the previous development of PE and, besides, related to a higher PE severity. The results obtained in the present work corroborate certain aspects described in previous studies and provide a new perspective on the role of aPLs and endothelial dysfunction parameters in the context of PE.

The prevalence of classical aPLs in the low risk obstetric population is around 1%–9% [39,40,41], and around 11%–20% [42,43] in women with PE. These data suggest, although inconsistently, an association between the presence of these antibodies and obstetric morbidity. The relationship between aPLs and PE in women without evidence of autoimmune diseases has been pursued in last decades [39,42,44,45,46,47,48,49,50]. However, it remains unclear and different researchers have not found such an association [51,52,53,54]. On the other hand, it is well known that any inflammatory phenomenon can be an inducer of aPL [55,56], so it cannot be ruled out that the inflammatory state associated with PE could have induced the production of aPL and that the finding of these antibodies three months after delivery could be the result of reverse causality.

While most of the studies that have analyzed the relationship between aPLs and obstetric complications of APS, or PE, have focused on the antibodies included as diagnosis criteria for APS (AL, aCL or aβ2GPI) and mainly on the estimation of IgG and IgM isotypes, only a few studies have analyzed the pathogenic importance of the IgA isotype of aPLs [57,58].

Several studies indicate that the positivity for IgA aβ2GPI antibodies is more strongly associated with recurrent miscarriages and unexplained fetal deaths than IgA aCL antibodies in women who show negative results for AL and IgG and IgM aCL isotypes. A possible explanation for this observation is that the correlation between IgA aβ2GPI and IgA aCL is weaker than the correlation between the IgG and IgM isotypes of these antibodies [57,58].

The presence of aPS/PT has been reported as a high risk factor for thrombosis [59] and its combination with AL and aβ2GPI, can be useful to identify subgroups of patients at risk for thromboembolic events [60]. Various studies have demonstrated the clinical utility of aPS/PT in the diagnosis of APS, especially in the absence of antibodies included in the diagnostic criteria [15,61,62]. Likewise, several studies have indicated a strong association between aPS/PT antibodies and obstetric morbidity [20,59,63]. However, they have not yet been included as classification criteria for APS because some drawbacks in the studies, such as the small size on which these data are based, the methodological differences in their design, the lack of standardization in their determination and the contradictory results related to their association with the APS.

The results of our study show that among the patients who developed PE, the prevalence of classical antibodies was 18.9%, being IgG aβ2GPI the most prevalent among them (13.9%), while the prevalence of unconventional antibodies was around four times higher than classical antibodies (54.9%), with IgM aPS/PT as the most prevalent antibody (47.5%). Unlike previous studies [64], we have not found any significant correlation between IgG/IgM aPS/PT with any of the classical antibodies. The fact that our patients did not present symptoms as severe as those observed in the OAPS, could justify this lack of correlation. Regarding the association between aPL and PE, we have not found an association between aCL, aβ2GPI or AL with PE, which is in agreement with previous studies. Thus Abou-Nassar et al. [65], showed that AL and aß2GPI are much more associated with thrombotic than with obstetric events, while aCL and aPS/PT seem to be associated with both types of complications. In another systematic review and meta-analysis, do Prado et al. [66] observed that moderate-high aCL titers are associated with PE, although they did not find evidence enough to use this type of antibody as predictor of PE in clinical practice. Regarding unconventional antibodies, our results are in agreement with those obtained by Žigon et al. [23], who indicate that aPS/PT are a stronger independent risk factor for obstetric complications compared to AL, aCL and aß2GPI.

The mechanisms of the increased cardiovascular risk among women with a history of PE [67,68] are not well understood. An endothelial dysfuntion has been observed in both PE and atherosclerosis, providing a plausible link between these two conditions [69], whose non-invasive evaluation can be carried out using PWV [70] and the ABI [71]. In normal pregnancy, several hemodynamic adaptations take place involving a decrease in PWV during the first and second trimester of gestation, increasing from the beginning of the third trimester to immediately after delivery, and decreasing to similar levels at the beginning of pregnancy approximately one month later childbirth [34,72]. Conversely, women with PE show a significant increase in PWV throughout the three trimesters of pregnancy, which persists even after delivery [35] and suggests that patients with PE have a higher cardiovascular risk after delivery. A systematic review shows that preeclampsia is associated with increased arterial stiffness during and after pregnancy, as well as the existence of an association between the severity of preeclampsia and arterial stiffness [73,74]. After adjusting for possible confounding factors, a significant and inverse correlation was found between PWV and global endothelial function in healthy patients (r = 0.69; *p* = 0.001). A systematic review by Cecelja et al. [75] found that, despite the exception of age and hypertension, PWV is practically independent of the classical risk factors for atherosclerosis. Our results confirm these data because at the time of PWV evaluation, the patients included in the study were already not hypertensive, so that, in addition to positivity in IgM aPS/PT, we only found age as a factor associated with altered PWV values.

The ABI is an objective and recognized method for the evaluation of peripheral vascular function in asymptomatic people. An ABI value < 0.9 is a good predictor of cardiovascular morbidity and mortality related to peripheral arterial disease, with a sensitivity of 95% and a specificity of almost 100% for detecting the disease by angiographic methods [71]. A comprehensive systematic review and meta-analysis [76] found that the 10-year cardiovascular mortality in women with a low ABI (≤0.9) was 12.6% (95% CI, 6.2%–19.0%) and 4.1% (95% CI, 2.2%–6.1%) in those women with a normal ABI (HR, 3.5; 95% CI, 2.4–5.1). It is possible that the transient but severe endothelial dysfunction seen in PE potentiates a cascade of events that progresses to atherosclerosis [68]. However, despite the fact that in our study we have only found age and the positivity of IgM aPS/PT as factors associated with subclinical atherosclerosis, it does not seem that they are the only parameters responsible for it. In our study, we found a high positive correlation between IgM aPTPS and PWV levels (ρ = 0.830; *p* < 0.001), as well as a moderate inverse correlation with the ABI (ρ = −0.469; *p* < 0.001).

The analysis of the vascular parameters showed that those patients with normal pregnancies and negative IgM aPS/PT titers presented lower PWV levels in comparison with women that had IgM aPS/PT antibodies (PWV 7.50 (7.20–8.00) m/s vs. 9.00 (8.35–9.95) m/s; *p* < 0.001). However, ABI was similar between both groups of patients. Furthermore, significant differences in PWV (7.80 (7.10–8.00) m/s vs. 9.50 (8.88–10.70) m/s; *p* < 0.001) and ABI (1.11 (0.95–1.22) vs 0.90 (0.86–1.14); *p* < 0.001) were observed between those women who developed PE and presented IgM aPS/PT in comparison with PE women without IgM aPS/PT, which suggest that endothelial dysfunction in preeclamptic women may be significantly accentuated in the presence of IgM aPS/PT.

Finally, regarding traditional risk factors for PE, we identified an association between family history of hypertension and the development of PE (OR maternal = 3.47; IC 95% (1.34–8.98); *p* = 0.010) and (OR paternal = 3.06; IC 95% (1.29–7.27); *p* = 0.011). However, only maternal family history of hypertension was included in the final multivariate regression analysis, showing an intense association OR = 8.3 (1.2–57.4), *p* = 0.032. These results confirm the contribution of the familial component in the appearance of PE [77]. Nevertheless, we did not observe any association with other classically associated risk factors. In this way, despite 12 of 16 women included in the study with an age above 40 years developed PE, we did not find a significant increase in the risk of PE as a consequence of the advanced age. This could be explained because all the women were under 44 years old. These results are in agreement with the results obtained by Lehman [78], who did not find a clear increase in the incidence of PE until the women reached 45 years old. Although the use of assisted reproductive techniques has been associated with the development of PE, especially in the case of multiple pregnancies [79], we did not observe any association. Finally, due to the small number of patients with the following antecedents, we could not confirm the increased risk of PE derived from a history of PE in previous pregnancies [80], the presence of urinary tract infections [81], or the protective effect of smoking in the development of PE [82].

As main weaknesses of our study, we highlight the small size of the control group compared to the PD group, as well as the absence of determination of aPL and cardiovascular parameters during pregnancy. In future studies, we should analyze whether unconventional APLs already develop in the first months of pregnancy and could be a pathogenic factor of this disease.

In conclusion, although its clinical relevance and its mechanisms of action are far from being well understood, the observed results suggest that in those patients who have developed PE, the presence of IgM aPS/PT could be useful for the stratification of CV risk, helping to identify those women who could benefit from an adequate therapeutic treatment. However, multicenter studies are required to validate the independent effects of these antibodies and, where appropriate, to confirm their usefulness in clinical practice.

## 4. Materials and Methods

### 4.1. Patients

The patients of this study included 157 pregnant women with a high risk of PE, defined as one or more of the following criteria: previous PE, chronic kidney disease, diabetes mellitus (pre-pregnancy), pregnancy by assisted reproduction techniques, maternal age > 40 years, BMI > 35 Kg/m^2^ at the beginning of pregnancy; multiple pregnancy or familial history of PE were included in the present study. Further, 122 (77.71%) women developed PE (56 non-severe PE (NS-PE) and 66 severe PE (S-PE)). The gestation proceeded without hypertensive disorders of pregnancy in the remaining 35 women.

The main demographic data, as well as personal, familial and obstetric history were collected using an individualized questionnaire supplemented with the patient’s clinical history. Pregnancy and analytical data were obtained from records incorporated into the clinical history. The study was conducted following the rules of Declaration of Helsinki and approved by the Regional Ethics Committee in our Institution (reference number: 2018.170). All the patients included in the present study gave informed consent before inclusion in the study. Diagnostic criteria for PE include a systolic blood pressure of 140 mm Hg or more or diastolic blood pressure of 90 mm Hg or more on two occasions at least 4 h apart after 20 weeks of gestation in a woman with a previously normal blood pressure and proteinuria of 300 mg or more per 24 h urine collection (or this amount extrapolated from a timed collection) or protein/creatinine ratio of 0.3 mg/dL or more or dipstick reading of 2+ (used only if other quantitative methods not available). Preeclampsia with severe features incluide systolic blood pressure of 160 mm Hg or more, or diastolic blood pressure of 110 mm Hg or more on two occasions at least 4 h apart (unless antihypertensive therapy is initiated before this time), thrombocytopenia (platelet count less than 100 × 109/L, impaired liver function that is not accounted for by alternative diagnoses and as indicated by abnormally elevated blood concentrations of liver enzymes (to more than twice the upper limit normal concentrations), or by severe persistent right upper quadrant or epigastric pain unresponsive to medications, renal insufficiency (serum creatinine concentration more than 1.1 mg/dL or a doubling of the serum creatinine concentration in the absence of other renal disease), pulmonary edema, new-onset headache unresponsive to medication and not accounted for by alternative diagnosesor visual disturbances. The clinical criteria used for the diagnosis and classification of PE were reviewed, checking their adjustment to guidelines established by The American College of Obstetricians and Gynecologists (ACOG, Task Force on Hypertension in Pregnancy) [1].

The determination of the classical and unconventional aPLs together with the vascular parameters (PWV and ABI) was performed at three months after delivery, once solved the hypertension and the proteinuria in those patients with PE during pregnancy.

### 4.2. ELISA Serum Antiphospholipid Antibodies

Ten ml of peripheral blood was collected at three months postpartum in a tube without anticoagulant. The serum was extracted by centrifugation at 3000 revolutions per minute during 10 min at room temperature.

This serum was used to determine the presence of aPLs by ELISA following manufacturer instructions. Specifically, IgG and IgM aCL and IgG and IgM aβ2GPI were measured using ORGENTEC detection kit (Diagnostika GmbH^®^, Straßberg, Germany) (ref ORG 515 y 521, respectively). Additionally, IgA aCL (ref 708635), IgA aβ2GPI (ref 708675), and IgG and IgM aPS/PT (ref 708835 and 708845, respectively) were detected using QUANTA Lite^®^ (Inova Diagnostics, Inc., San Diego, CA, USA). The cut-off points established to determine the positivity of the different aPLs are depicted in Supplementary Table S3.

### 4.3. Lupus Anticoagulant Test

Activated Partial Thromboplastin Time (APTT)-based mixing test and Russell Viper Venom Time (RVVT) test were used to determine lupus anticoagulant.

### 4.4. PWV and ABI Determination

The PWV was determined using the automated device SphymoCor XCEL and the ABI was measured using the WatchBP Office ABI system (Microfile whatchBP AG, Widnau, Switzerland).

PWV values were considered abnormal if they exceeded the median reference value of the patient’s age group, established for the European population [83] (Supplementary Table S2). Abnormal ABI values were considered those less than 0.9.

### 4.5. Statistical Analysis

Statistical analysis was performed using SPSS v.15.0 (IBM Corp., Armonk, NY, USA). The distribution of continuous variables was assessed using Kolmogorov–Smirnov/Shapiro–Wilk tests where indicated. Results were expressed as mean ± standard deviation or median + interquartile range (IQR) for continuous variables and percentages for categorical data. Comparisons were based on the chi squared test or Fisher´s exact test for categorical data and Mann–Whitney U test for nonparametric continuous data. Receiver operating characteristic (ROC) analysis and Youden’s index were used to determine the optimal cut-point with higher sensitivity and specificity of each aPL for the development of PE. Spearman rank correlation was used to quantify the association between aPL titers and cardiovascular parameters (PWV and ABI) considering both as continuous variables. In the multivariate logistic regression analysis the Backward Stepwise procedure was used after selecting the variables identified by the univariate models as potential predictors, as well as those others whose presence in the model was methodologically justified. Only variables considered statistically significant (*p* < 0.05) were included in the final model.

## Figures and Tables

**Table 1:** Prevalence of Antiphospholipid Antibodies.

	Analysis Group				Severity of PE		
	Total n = 157	Control n = 35	PE n = 122	*p*	NS-PE n = 56	S-PE n = 66	*p*
**Classical aPLs**	31 (19.7%)	8 (22.9%)	23 (18.9%)	0.600	8 (14.3%)	15 (22.7%)	0.235
LA	5 (3.2%)	2 (5.7%)	3 (2.5%)	0.309	2 (3.6%)	1 (1.5%)	0.593
IgG aCL	9 (5.7%)	3 (8.6%)	6 (4.9%)	0.418	2 (3.6%)	4 (6.1%)	0.686
IgM aCL	6 (3.8%)	1 (2.9%)	5 (4.1%)	0.736	−	5 (7.6%)	−
IgG aβ2GPI	19 (12.1%)	2 (5.7%)	17 (13.9%)	0.248	6 (10.7%)	11 (16.7%)	0.344
IgM aβ2GPI	14 (8.9%)	3 (8.6%)	11 (9.0%)	0.935	5 (8.9%)	6 (9.1%)	0.975
**Unconventional aPLs**	75 (47.8%)	8 (22.9%)	67 (54.9%)	0.001	21 (37.5%)	46 (69.7%)	<0.001
IgA aCL	4 (2.5%)	−	4 (3.3%)	−	1 (1.8%)	3 (4.5%)	0.624
IgA aβ2GPI	10 (6.4%)	1 (2.9%)	9 (7.4%)	0.460	2 (3.6%)	7 (10.6%)	0.177
IgG aPS/PT	11 (7.0%)	1 (2.9%)	10 (8.2%)	0.458	4 (7.1%)	6 (9.1%)	0.752
IgM aPS/PT	63 (40.1%)	5 (14.3%)	58 (47.5%)	<0.001	16 (28.6%)	42 (63.6)	<0.001

PE: pre-eclampsia; NS-PE: non-severe pre-eclampsia; S-PE: severe pre-eclampsia; aPL: antiphospholipid antibodies; LA: lupus anticoagulant; aCL: anticardiolipin; aβ2GPI: anti-β2-glycoprotein I; aPS/PT: antiphosphatidylserine/pro-thrombin.

**Table 2:** Association Between Antiphospholipid Antibodies and pre-Eclampsia Severity.

	PE OR (CI 95%)	*p*	S-PE OR (CI 95%)	*p*
**Classical aPLs**	**0.8 (0.3–1.9)**	**0.600**	**1.8 (0.7** **–** **4.5)**	**0.238**
LA	0.4 (0.1–2.6)	0.348	0.4 (0.0–4.7)	0.478
IgG aCL	0.6 (0.1–2.3)	0.418	1.7 (0.3–9.9)	0.531
IgM aCL	1.5 (0.2–12.9)	0.737	−	−
IgG aβ2GPI	2.7 (0.6–12.2)	0.204	1.7 (0.6–4.8)	0.348
IgM aβ2GPI	1.1 (0.3–4.0)	0.935	1.0 (0.3–3.5)	0.975
**Unconventional aPLs**	**4.1 (1.7–9.8)**	**0.001**	**3.8 (1.8–8.1)**	**<0.001**
IgA aCL	−	−	2.6 (0.3–25.9)	0.410
IgA aβ2GPI	2.7 (0.3–22.1)	0.353	3.2 (0.6–16.1)	0.157
IgG aPS/PT	3.0 (0.4–24.6)	0.298	1.3 (0.3–4.9)	0.697
IgM aPS/PT	5.4 (2.0–14.9)	0.001	4.4 (2.0–9.4)	<0.001

PE: pre-eclampsia; S-PE: severe pre-eclampsia; CI: confidence interval; aPLs: antiphospholipid antibodies; LA: lupus anticoagulant; aCL: anticardiolipin; aβ2GPI: anti-β2-glycoprotein I; aPS/PT: antiphosphatidylserine/prothrombin.

**Table 3:** Cardiovascular Parameters in Women with PE 3 Months after Delivery.

	Analysis Group			Severity of PE		
	Control n = 35	PE n = 122	*p*	NS-PE n = 56	S-PE n = 66	*p*
**BMI (Kg/m^2^)**	23.1 (20.0–27.8)	27.0 (24.2–30.0)	0.001	27.9 (24.2–30.9)	26.8 (24.1–29.5)	0.342
**SBP (mm Hg)**	122.0 (112.0–129.0)	120.0 (111.0–126.0)	0.166	121.0 (111.0–128.0)	119.0 (110.8–124.0)	0.256
**DBP (mm Hg)**	72.0 (71.0–80.0)	76.0 (71.0–84.0)	0.132	76.0 (70.3–82.8)	77.0 (71.8–85.0)	0.666
**PWV (m/s)**	7.7 (7.2–8.0)	8.2 (7.6–9.5)	<0.001	8.0 (7.6–8.9)	9.0 (7.8–10.2)	0.015
**ABI**	1.16 (1.00–1.30)	1.04 (0.90–1.26)	<0.001	1.11 (0.97–1.36)	0.94 (0.89–1.24)	<0.001

PE: pre-eclampsia; NS-PE: non-severe pre-eclampsia; S-PE: severe pre-eclampsia; BMI: body mass index, SBP: systolic blood pressure; DBP: diastolic blood pressure; PWV: pulse wave velocity; ABI: ankle-brachial index.

**Table 4:** Parameters Associated with Cardiovascular Alterations in PE group.

	Altered PWV				Altered ABI			
	Univariate Analysis		Multivariate Analysis		Univariate Analysis		Multivariate Analysis	
	OR (CI 95%)	*p*	OR (CI 95%)	*p*	OR (CI 95%)	*p*	OR (CI 95%)	*p*
***Cardiovascular risk factors***								
Age	0.82 (0.66–1.02)	0.073	0.75 (0.54–1.05)	0.098	1.00 (0.93–1.10)	0.841	1.04 (0.95–1.12)	0.412
BMI	1.03 (0.89–1.18)	0.720	1.05 (0.87–1.28)	0.608	1.01 (0.93–1.10)	0.762	1.01 (0.94–1.10)	0.723
Diabetes	0.14 (0.01–1.77)	0.130	0.21 (0.03–1.81)	0.158	1.25 (0.11–14.24)	0.857	1.18 (0.09–12.51)	0.726
Smoking	0.86 (0.10–7.56)	0.894	0.82 (0.24–5.43)	0.921	3.21 (0.93–11.17)	0.066	0.98 (0.24–3.93)	0.976
**Unconventional aPLs**								
IgA aCL	1.04 (0.80–1.34)	0.782	0.92 (0.69–1.23)	0.560	1.00 (0.88–1.14)	0.990	0.97 (0.85–1.10)	0.623
IgA aβ2GPI	1.04 (0.89–1.21)	0.654	0.93 (0.78–1.13)	0.474	1.08 (1.01–1.15)	0.023	1.10 (1.02–1.18)	0.012
IgG aPS/PT	1.08 (0.92–1.27)	0.360	1.16 (0.88–1.53)	0.281	1.06 (1.00–1.14)	0.071	1.08 (1.00–1.16)	0.051
IgM aPS/PT	1.33 (1.10–1.59)	0.002	1.38 (1.10–1.73)	0.006	1.08 (1.04–1.13)	<0.001	1.09 (1.04–1.14)	<0.001

PE: pre-eclampsia; CI: confidence interval; PWV: pulse wave velocity; ABI: ankle-brachial index; BMI: body mass index; aPLs: antiphospholipid antibodies; aCL: anticardiolipin; aβ2GPI: antiβ2GPI; aPS/PT: antiphosphatidylserine/prothrombin.

## Supplementary Materials

The following are available online at https://www.mdpi.com/2075-1729/10/10/241/s1, Table S1: Personal, Family and Obstetric History. Pregnancy Data, Table S2: PWV Reference Values, Table S3: Cut-off Points Established for the Different Antiphospholipid Antibodies.

## Abbreviations

aβ2GPI	Antiβ2glycoprotein-I antibodies
ABI	Ankle-brachial index
aCL	Anticardiolipin antibodies
aPL	Antiphospholipid antibodies
APS	Antiphospholipid syndrome
aPS/PT	Antiphosphatidylserine-prothrombin complex antibodies
aPT	Antiprothrombin antibodies
APTT	Activated Partial Thromboplastin Time
BMI	Body mass index
DBP	Diastolic blood pressure
LA	Lupus anticoagulant
NS-PE	Non-severe pre-eclampsia
OAPS	Obstetric antiphospholipid syndrome
PE	Pre-eclampsia
PWV	Pulse wave velocity
RVVT	Russell Viper Venom Time
SBP	Systolic blood pressure
S-PE	Severe pre-eclampsia
