# Supplementary material for: Non-Criteria Antiphospholipid Antibodies: Risk Factors for Endothelial Dysfunction in Women with Pre-Eclampsia
Source: Life (Basel). 2020 Oct 14;10(10):241. doi: 10.3390/life10100241 (PMC7650548; doi:10.3390/life10100241)
Supplement: Supplementary file 1 [file life-10-00241-s001.zip › life-949419-supplementary.docx]

**Supplementary Table S1.** Personal, Family and Obstetric History. Pregnancy Data.

|  | **Controls**  **n = 35** | **PE**  **n = 122** | | ***p*** | |  |
| --- | --- | --- | --- | --- | --- | --- |
| **Previous to pregnancy cardiovascular risk factors and cardiovascular history** | | | | | |  |
| Age (years) | 35.2 ± 5.1 | 34.7 ± 5.2 | | 0.641 | |  |
| Hypertension | − | − | | − | |  |
| Diabetes Mellitus | − | 3 (2.5%) | | − | |  |
| Dyslipemia | 4 (11.4%) | − | | − | |  |
| Smoking | 5 (14.3%) | 12 (9.8%) | | 0.455 | |  |
| Arterial or venous thrombosis | − | − | | − | |  |
| **Obstetric history** |  |  | |  | |  |
| Multiparity | 20 (57.1%) | 55 (45.1%) | | 0.251 | |  |
| Total pregnancy loss | 11 (31.4%) | 25 (20.5%) | | 0.092 | |  |
| Voluntary interruption | 8 (22.9%) | 10 (8.2%) | | 0.040 | |  |
| Gestational diabetes | 1 (2.9%) | 2 (1.6%) | | 0.533 | |  |
| **Familial history** |  |  | |  | |  |
| Mother |  |  | |  | |  |
| Hypertension | 6 (17.1%) | 51 (41.8%) | | 0.007 | |  |
| Diabetes Mellitus | 13 (37.1%) | 22 (18.0%) | | 0.017 | |  |
| Father |  |  | |  | |  |
| Hypertension | 8 (22.9%) | 58 (47.5%) | | 0.009 | |  |
| Diabetes Mellitus | 7 (20.0%) | 41 (33.6%) | | 0.124 | |  |
| **Current pregnancy** |  |  | |  | |  |
| Assisted reproductive techniques | 15 (42.9%) | 20 (16.4%) | | 0.002 | |  |
| Multiple gestation | 3 (8.6%) | 9 (7.4%) | | 0.815 | |  |
| Caesarean birth | 11 (31.4%) | 71 (58.2%) | | 0.005 | |  |
| Delivery week | 38.3 ± 1.9 | 36.6 ± 3.6 | | 0.008 | |  |
| **Newborn parameters** |  |  | |  | |  |
| APGAR | 8.7 ± 0.6 | 7,8 ± 1,2 | | 0.001 | |  |
| Newborn weight (g) | 3600.0  (3312.5−3777.5) | 2900.0  (2300.0−3407.5) | | < 0.001 | |  |
| Umbilical cord blood pH | 7.3 ± 0.1 | | 7.2 ± 0.1 | | 0.012 | |
| **Maternal complications** |  | |  | |  | |
| Eclampsia | − | | 2 (1.6%) | | − | |
| HELLP syndrome | − | | 16 (13.1%) | | − | |
| Hemorrhage | 1 (2.9%) | | 7 (5.7%) | | 0.685 | |
| Proteinuria | − | | 110 (90.2%) | | − | |
| Thrombopenia | 1 (2.9%) | | 44 (36.1%) | | <0.001 | |
| Altered liver function | − | | 24 (19.7%) | | − | |
| Altered kidney function | − | | 18 (14.8%) | | − | |

PE: pre-eclampsia.

**Supplementary Table S2.** PWV Reference Values.

| **Age (years)** | **Mean (± 2 SD)** | **Median (P10-P90)** |
| --- | --- | --- |
| < 30 | 6.2 (4.7–7.6) | 6.1 (5.3–7.1) |
| 30–39 | 6.5 (3.8–9.2) | 6.4 (5.2–8,0) |
| 40–49 | 7.2 (4.6–9.8) | 6.9 (5.9–8.6) |
| 50–59 | 8.3 (4.5–12.1) | 8.1 (6.3–10.0) |
| 60–69 | 10.3 (5.5–15.0) | 9.7 (7.9–13.1) |
| ≥ 70 | 10.9 (5.5–16.3) | 10.6 (8.0–14.6) |

SD: Standard deviation, P10: Percentile 10, P90: Percentile 90.

**Supplementary Table S3.** Cut-off Points Established for the Different Antiphospholipid Antibodies.

|  | **Negative** | **Positive** |
| --- | --- | --- |
| **Classical aPLs** |  |  |
| IgG aCL | < 6 GPL | ≥ 6 GPL |
| IgM aCL | < 6 MPL | ≥ 6 MPL |
| IgG aβ2GPI | < 4 U/mL | ≥ 4 U/mL |
| IgM aβ2GPI | < 4 U/mL | ≥ 4 U/mL |
| **Unconventional aPLs** |  |  |
| IgA aCL | < 12 APL | ≥ 12 APL |
| IgA aβ2GPI | < 12 APL | ≥ 12 APL |
| IgG aPS/PT | < 15 U/mL | ≥ 15 U/mL |
| IgM aPS/PT | < 15 U/mL | ≥ 15 U/mL |

aPL: antiphospholipid antibodies, aCL: anticardiolipin, aβ2GPI anti-β2-glycoprotein I,aPS/PT: antiphosphatidylserine/prothrombin.
